# Intracranial Rhabdomyoma: Case Report and Review of the Literature

**DOI:** 10.7759/cureus.593

**Published:** 2016-04-29

**Authors:** David R Santiago-Dieppa, Tianzan Zhou, Karra A Jones, Brandon C Gabel, James Y Chen, Lawrence Hansen, Hoi Sang U

**Affiliations:** 1 Department of Neurosurgery, University of California, San Diego; 2 Department of Pathology, University of California, San Diego; 3 Department of Radiology, University of California, San Diego

**Keywords:** rhabdomyoma, trigeminal nerve, neuromuscular choristoma, intracranial

## Abstract

A 24-year-old male presented with eight months of increasingly severe frontal headaches, decreased right facial sensation, and periodic vertigo. Magnetic resonance imaging demonstrated a heterogeneously contrast-enhancing mass involving and expanding the right foramen ovale.  A biopsy of the lesion was performed, and the final pathologic diagnosis revealed a neoplastic rhabdomyoma. To date, only five cases of intracranial rhabdomyoma have been reported, and a rhabdomyoma involving the trigeminal nerve has never been described in an adult. This manuscript reviews the available literature and highlights the clinical, imaging, pathologic characteristics, and surgical management of these exceedingly rare lesions.

## Introduction

Rhabdomyomas are rare neoplasms of skeletal muscle that typically arise in oral, cervical, vulvovaginal, and cardiac tissue [1]. In contrast, neuromuscular choristomas are rare hamartomatous lesions that are thought to be malformations that affect major peripheral nerve trunks [1]. Histologically, neuromuscular choristomas are characterized by intersecting myocyte fascicles interwoven amongst variably myelinated axons. Rhabdomyomas occurring in association with peripheral nerves can appear histologically similar to neuromuscular choristomas; they are distinguished from one another by their neoplastic and non-neoplastic classifications, respectively. 

We report the case of a 24-year-old male with a heterogeneously enhancing expansile mass within the right foramen ovale on magnetic resonance imaging. On pathologic examination, the lesion demonstrated histologic features of a neuromuscular choristoma, but with proliferating Ki-67-positive cells, which therefore supports the diagnosis of neoplastic rhabdomyoma.

## Case presentation

**Figure 1:**
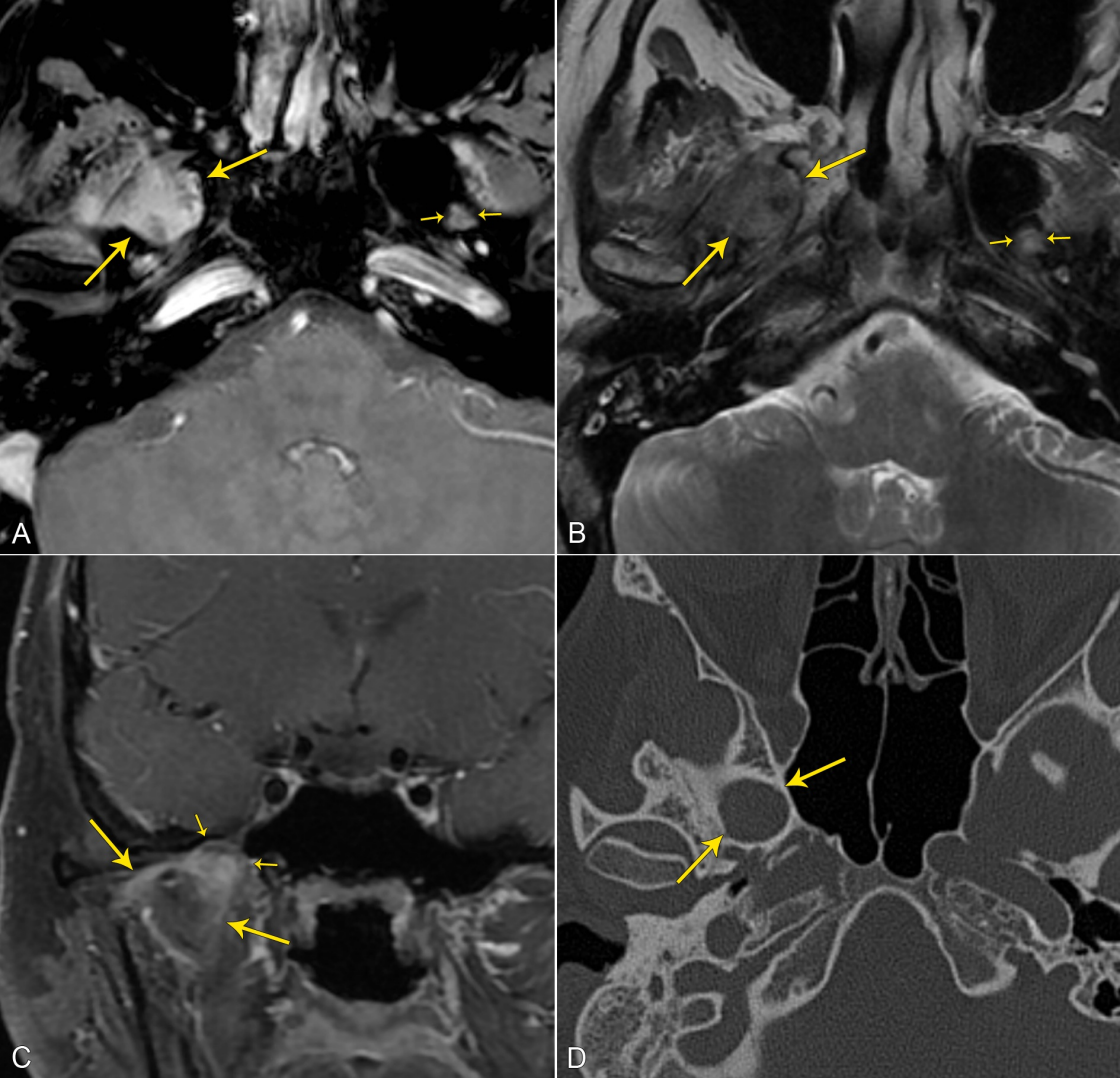
Preoperative Neuroimaging (A) Axial post-contrast fat-saturated T1-weighted MR image demonstrates a heterogeneously enhancing mass (long yellow-arrows) in the expanded right foramen of ovale. The normal left foramen of ovale is small by comparison (small yellow-arrows). (B) Axial T2-weighted MR image demonstrates the mildly heterogeneous mass (long yellow-arrows), predominantly isointense to gray matter, in the right foramen of ovale. The normal left foramen of ovale is small by comparison (small yellow-arrows). (C) Coronal post-contrast fat-saturated T1-weighted MR image demonstrates thin areas of linear enhancement (long yellow-arrows) extending into/infiltrating the muscles of mastication from the mass in the foramen ovale (short yellow-arrows). (D) Axial bone-window CT of the skull base demonstrates smooth, non-destructive expansion of the right foramen of ovale.

A 24-year-old male presented with an eight-month history of bilateral frontal headaches that were progressively increasing in frequency and severity. The patient endorsed intermittent vertigo. Neurologic examination revealed decreased sensation to light touch on the right forehead and cheek. MRI with and without contrast revealed a heterogeneously enhancing mass within the foramen ovale with surrounding edema and enhancement of the pterygoid muscles; computerized tomography of the head revealed a smooth, nondestructive expansion of the right foramen ovale (Figure 1). Informed patient consent was obtained prior to treatment.

A right subtemporal craniotomy was performed and exposure of the foramen ovale was achieved extradurally. This revealed the entire tumor, which involved the third segment of the trigeminal nerve and extended through the foramen ovale into the extracranial space. The lesion had the gross appearance of fibrous scar tissue.

**Figure 2:**
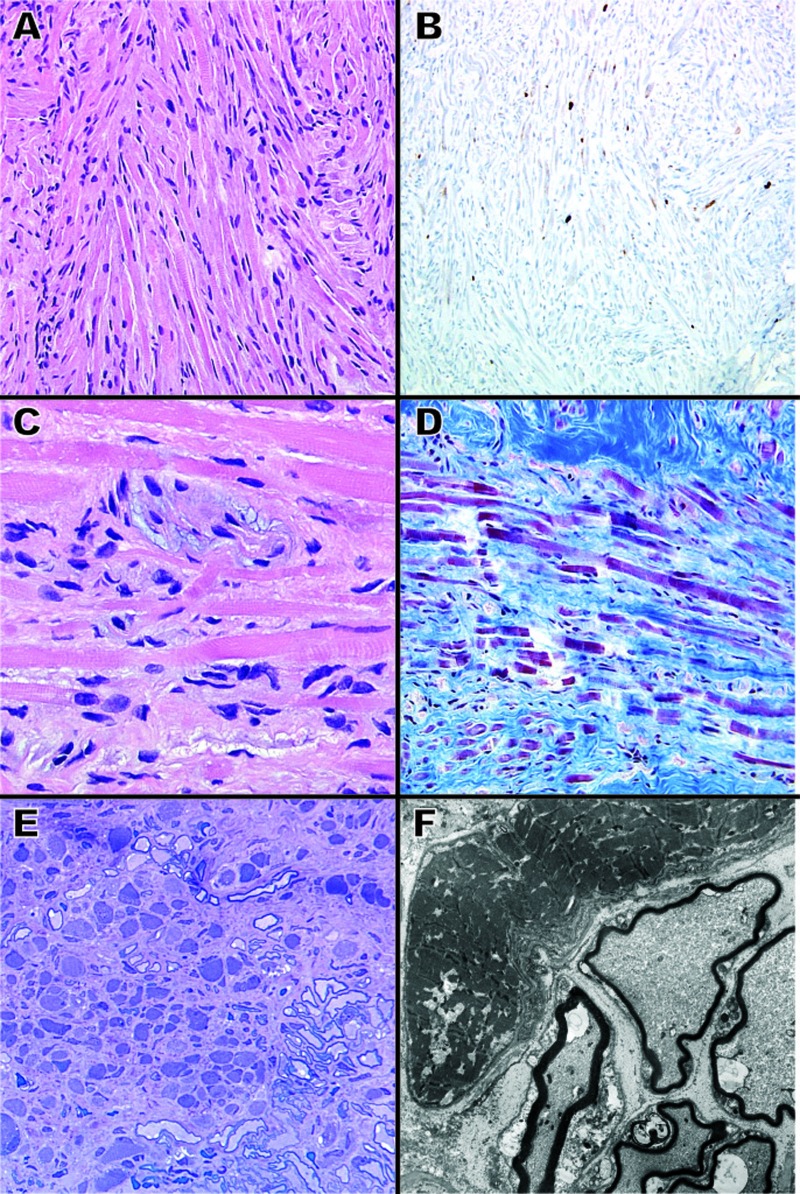
Histopathology (A) Photomicrograph showing interweaving fascicles of skeletal muscle cells with conspicuous striations (H&E; 200X original magnification). (B) Ki-67 immunohistochemical stain showing a few scattered proliferating fibers (200X original magnification). (C) Photomicrograph displaying peripheral nerve and myelin closely admixed within skeletal muscle fibers (H&E; 600X original magnification). (D) Trichrome histochemical stain highlighting skeletal muscle fibers in bright red, myelinated nerves shown by bubbly red/pink staining, and fibrous connective tissue in blue (200X original magnification). (E) One micron thick toluidine blue-stained section showing multiple small and irregular skeletal muscle fibers with intermixed myelinated nerve fibers (200X original magnification). (F) Electron microscopic image showing a relatively normal skeletal muscle fiber adjacent to multiple myelinated nerve fibers (4,000X original magnification).

Hematoxylin and eosin (H&E) stained specimens revealed disordered skeletal muscle fibers, peripheral nerve bundles, dense fibrous connective tissue, and scattered mature adipose tissue. Intersecting and interwoven fascicles of myocytes, ranging from elongate strap cells to rotund globular forms, were observed. Figures 2A-2C demonstrate the atypical orientation of the muscle and nerve fibers. Running within and alongside the muscle fibers were many variably myelinated nerve fibers. A Masson’s trichrome preparation highlighted the red skeletal muscle fibers set within a blue connective tissue background interrupted by peripheral nerve with light red myelin (Figure 2D). Toluidine blue-stained one-micron thick resin sections revealed well-differentiated skeletal muscle tissue that was organized into single myofibers that appeared to be largely independent of each other (Figure 2E). As shown, the myofibers were separated by well-differentiated and highly developed peripheral nerve myelinated axons (Figure 2E).

Electron microscopy examination confirmed the presence of both well-differentiated skeletal muscle cells and highly differentiated but thinly myelinated peripheral nerve (Figure 2F). The muscle cells contained actin and myosin-type filaments, but some myofibers demonstrated abnormal degeneration. The peripheral nerves showed degenerative changes as well as inclusion-like material.

Finally, immunohistochemistry for Ki-67 (30-9, Ventana, pre-dilute) was performed to determine if the lesion was, in fact, proliferating. As shown in Figure 2B, the neoplasm contained positivity for Ki-67 with up to 1-2% nuclear positivity.

The patient did well after surgery with no new postoperative neurologic deficits. Relief of facial pain was achieved. He has remained free of tumor progression on serial MRI imaging for over 18 months of follow-up.

## Discussion

The finding of benign skeletal muscle fibers admixed with myelinated axons and adipose tissue suggests a diagnosis of neuromuscular choristoma/hamartoma, rhabdomyoma, or myolipoma. In this patient, the predominate histopathology was intermingling benign muscle and nerve. Thus, a differential of neuromuscular choristoma versus rhabdomyoma is favored. Neuromuscular choristomas are hamartomatous lesions that have been reported to occur in cranial nerves, including the trigeminal nerve, but this is rare [1-2]. In fact, extracardiac rhabdomyomas of the cranial nerves are considered neoplastic.

While the literature suggests that lesions without a significant neural component are favored to be rhabdomyomas over choristomas [1], we prefer to categorize these lesions primarily by their neoplastic versus hamartomatous nature. Furthermore, four of the five previously reported intracranial rhabdomyomas were diagnosed before the common use of the Ki-67 proliferation index [3-5]. Interestingly, in this case, the predominant histology seen here was most consistent with a neuromuscular choristoma, but we performed immunohistochemistry for Ki-67 to determine if this lesion was, in fact, proliferating. As seen in Figure 2B, the neoplasm showed variable positivity for Ki-67 with areas showing up to 1-2% nuclear positivity, which is in agreement with previously reported values for intracranial rhabdomyomas [1]. This finding supports the diagnosis of a trigeminal nerve rhabdomyoma.

Little is known about the prognosis of intracranial rhabdomyomas. Vandewalle, et al. reported the case of a 41-year-old Caucasian male who suffered from progressive paresis of the right facial nerve since childhood [2]. The patient eventually presented for neurosurgical evaluation after he developed two years of progressive hearing loss. MRI revealed an 8 x 10 mm mass emerging from the right porus acusticus. A posterior translabyrinthine craniotomy for gross total resection and sacrifice of the facial nerve was performed. Thus, as illustrated by this case, it can be inferred that the progressive facial paralysis and eventual hearing loss observed in this patient was likely secondary to slow progression of the rhabdomyoma over several decades.

There is currently no consensus on the optimal treatment of intracranial rhabdomyomas. Van Leeuwen, et al. reported the case of a six-year-old male who presented with several years of progressive hearing loss [4]. An MRI scan revealed a 17 mm CP-angle mass involving cranial nerve eight. A suboccipital craniotomy was performed for the subtotal resection of the mass, and the patient had no evidence of progression over four years of follow-up. In contrast, Harder, et al. described the case of a 68-year-old male who presented with tinnitus of the left ear [1]. The patient was found to have a CP-angle tumor exerting mild brainstem compression. He underwent a subtotal resection of the rhabdomyoma and was also without progression until a four-year screening MRI revealed recurrence. The patient was followed conservatively for an additional four years until he began to develop increasing vestibular symptoms at which point he underwent repeat decompression.

Since patients almost exclusively present with neurologic deficits, a strong case can be made for mechanical decompression of involved neurologic structures. Given the slow natural progression of the disease as evidenced by the available six cases, restoration and preservation of neurologic function, rather than maximal resection at the risk of neurologic compromise, should remain the neurosurgeon's primary goal (Table 1).

**Table 1:** Documented Cases of Intracranial Rhabdomyomas The following table includes the six known cases of intracranial rhabdomyomas, which includes our case. CN = cranial nerve; GTR = gross total resection; STR = subtotal resection

Author/Year	Age/Sex	Intracranial location	Treatment	Follow-up
Zwick, et al., 1989 [5]	2, M	CN V	Left temporoparietal craniotomy for GTR	No tumor progression at one year
Van Leeuwen, et al., 1995 [3]	6, M	CN VIII	Suboccipital craniotomy for STR	No tumor progression at four years
Vandewalle, et al., 1995 [4]	41, M	CN VII	Posterior translabyrinthine craniotomy for GTR	Not reported
Lee, et al., 2000 [2]	15, M	CN III	Pterional/anterior temporal craniotomy for STR	Intracranial hemorrhage at tumor resection site 3 months post-op, resulting in death
Harder, et al., 2013 [1]	68, M	CN VIII	Retrosigmoid craniotomy for STR	Evidence of recurrence on 4-year MRI, repeat resection 8 years post-op after onset of vestibular symptoms
Current case, 2014	24, M	CN V	Subtemporal craniotomy for biopsy and subtotal resection	No progression at 18 months

## Conclusions

Neurosurgeons should understand the natural progression and clinical behavior of intracranial rhabdomyomas. Nuclear proliferation indices, such as the Ki-67 immunostain, are important in differentiating intracranial rhabdomyomas from the more benign neuromuscular choristoma. Surgical management aimed at decompressing, restoring, and preserving neurologic function should remain the goal because these neoplasms demonstrate an indolent clinical progression.
